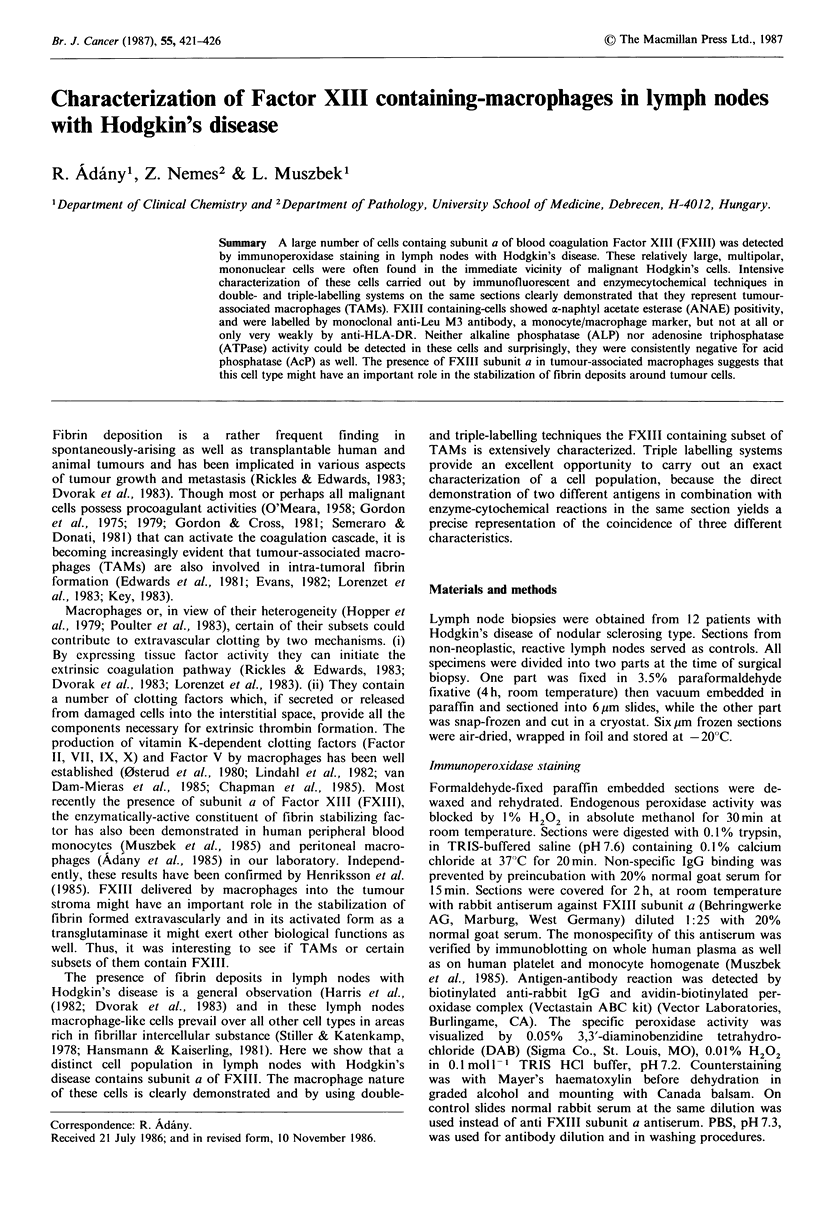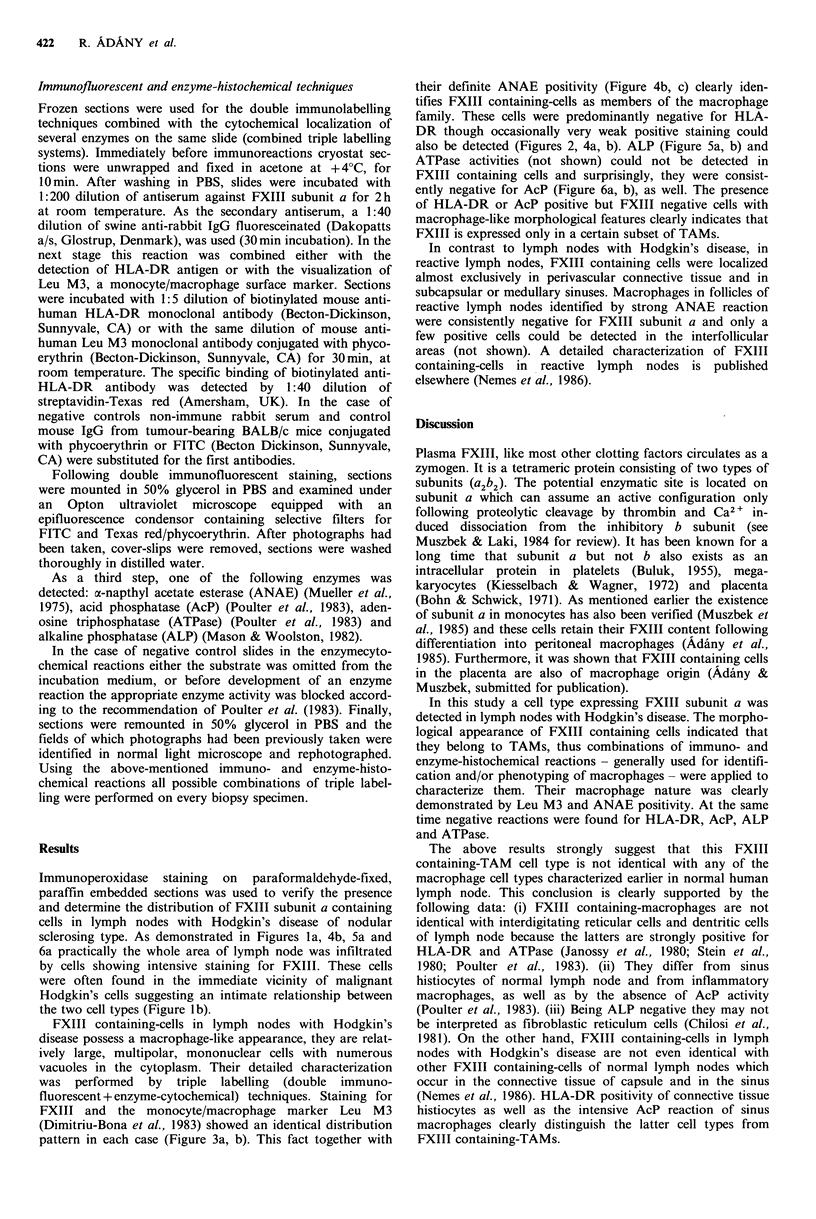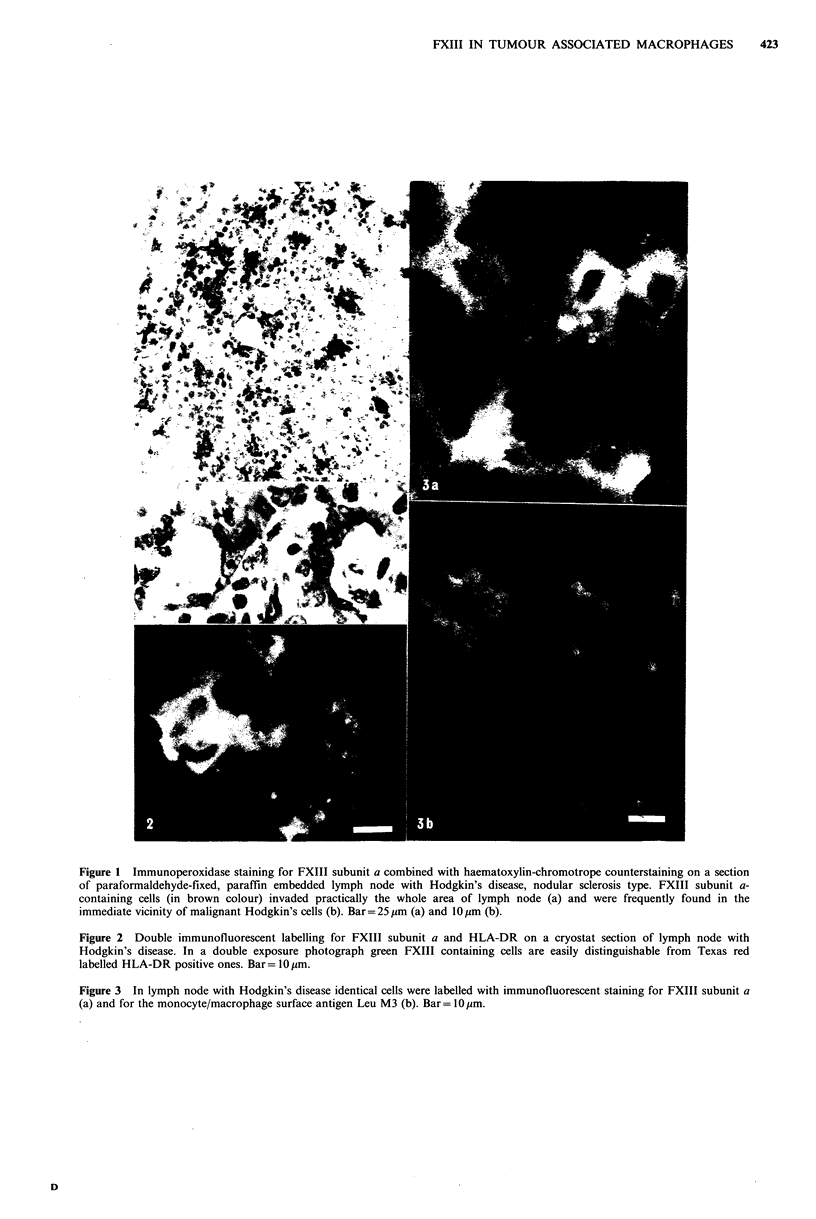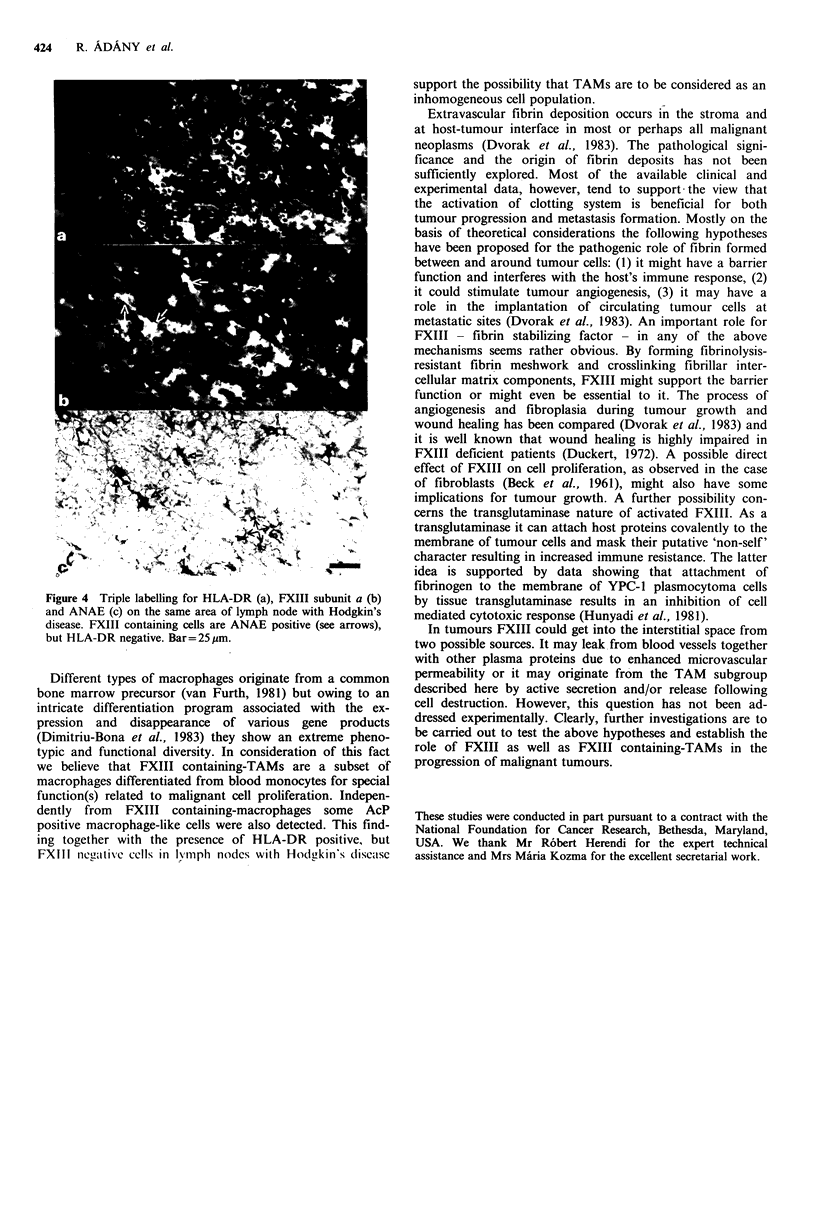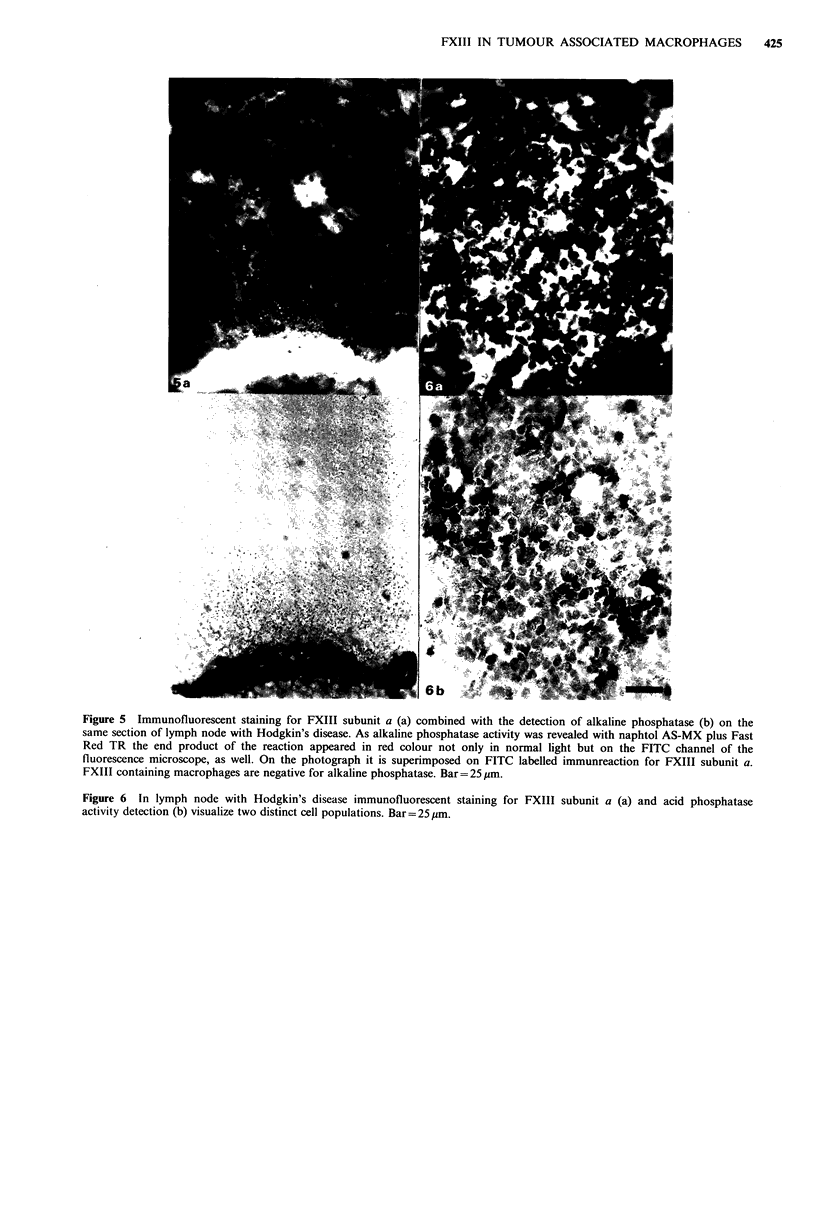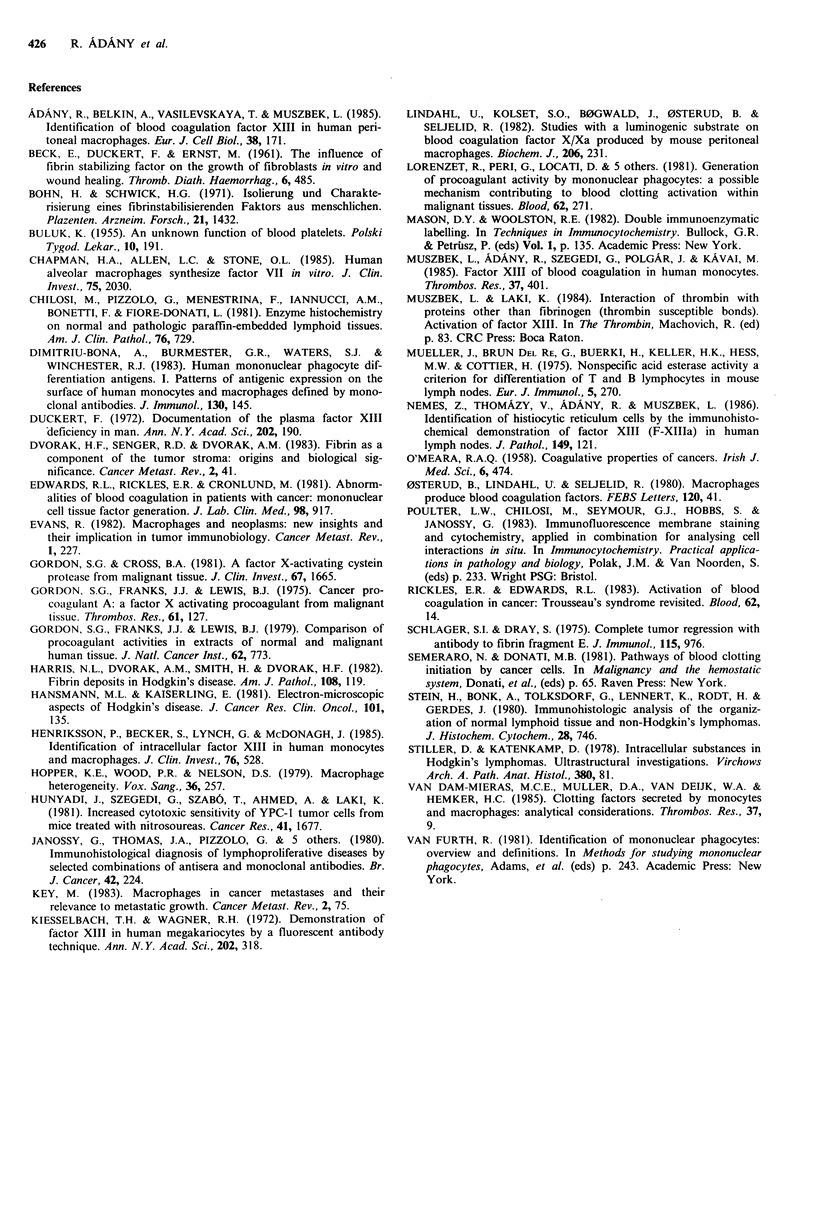# Characterization of factor XIII containing-macrophages in lymph nodes with Hodgkin's disease.

**DOI:** 10.1038/bjc.1987.82

**Published:** 1987-04

**Authors:** R. Adány, Z. Nemes, L. Muszbek

## Abstract

**Images:**


*Br. J. Cancer* (1987), **55**, 421–426 © The Macmillan Press Ltd., 1987

### Characterization of Factor XIII containing-macrophages in lymph nodes with Hodgkin's disease

R. Ádány[1], Z. Nemes[2] & L. Muszbek[1]

[1]*Department of Clinical Chemistry and* [2]*Department of Pathology, University School of Medicine, Debrecen, H-4012, Hungary.*

**Summary** A large number of cells containg subunit *a* of blood coagulation Factor XIII (FXIII) was detected by immunoperoxidase staining in lymph nodes with Hodgkin's disease. These relatively large, multipolar, mononuclear cells were often found in the immediate vicinity of malignant Hodgkin's cells. Intensive characterization of these cells carried out by immunofluorescent and enzymecytochemical techniques in double- and triple-labelling systems on the same sections clearly demonstrated that they represent tumour-associated macrophages (TAMs). FXIII containing-cells showed α-naphtyl acetate esterase (ANAE) positivity, and were labelled by monoclonal anti-Leu M3 antibody, a monocyte/macrophage marker, but not at all or only very weakly by anti-HLA-DR. Neither alkaline phosphatase (ALP) nor adenosine triphosphatase (ATPase) activity could be detected in these cells and surprisingly, they were consistently negative for acid phosphatase (AcP) as well. The presence of FXIII subunit *a* in tumour-associated macrophages suggests that this cell type might have an important role in the stabilization of fibrin deposits around tumour cells.

Fibrin deposition is a rather frequent finding in spontaneously-arising as well as transplantable human and animal tumours and has been implicated in various aspects of tumour growth and metastasis (Rickles & Edwards, 1983; Dvorak *et al.*, 1983). Though most or perhaps all malignant cells possess procoagulant activities (O'Meara, 1958; Gordon *et al.*, 1975; 1979; Gordon & Cross, 1981; Semeraro & Donati, 1981) that can activate the coagulation cascade, it is becoming increasingly evident that tumour-associated macrophages (TAMs) are also involved in intra-tumoral fibrin formation (Edwards *et al.*, 1981; Evans, 1982; Lorenzet *et al.*, 1983; Key, 1983).

Macrophages or, in view of their heterogeneity (Hopper *et al.*, 1979; Poulter *et al.*, 1983), certain of their subsets could contribute to extravascular clotting by two mechanisms. (i) By expressing tissue factor activity they can initiate the extrinsic coagulation pathway (Rickles & Edwards, 1983; Dvorak *et al.*, 1983; Lorenzet *et al.*, 1983). (ii) They contain a number of clotting factors which, if secreted or released from damaged cells into the interstitial space, provide all the components necessary for extrinsic thrombin formation. The production of vitamin K-dependent clotting factors (Factor II, VII, IX, X) and Factor V by macrophages has been well established (Østerud *et al.*, 1980; Lindahl *et al.*, 1982; van Dam-Mieras *et al.*, 1985; Chapman *et al.*, 1985). Most recently the presence of subunit *a* of Factor XIII (FXIII), the enzymatically-active constituent of fibrin stabilizing factor has also been demonstrated in human peripheral blood monocytes (Muszbek *et al.*, 1985) and peritoneal macrophages (Ádány *et al.*, 1985) in our laboratory. Independently, these results have been confirmed by Henriksson *et al.* (1985). FXIII delivered by macrophages into the tumour stroma might have an important role in the stabilization of fibrin formed extravascularly and in its activated form as a transglutaminase it might exert other biological functions as well. Thus, it was interesting to see if TAMs or certain subsets of them contain FXIII.

The presence of fibrin deposits in lymph nodes with Hodgkin's disease is a general observation (Harris *et al.*, (1982; Dvorak *et al.*, 1983) and in these lymph nodes macrophage-like cells prevail over all other cell types in areas rich in fibrillar intercellular substance (Stiller & Katenkamp, 1978; Hansmann & Kaiserling, 1981). Here we show that a distinct cell population in lymph nodes with Hodgkin's disease contains subunit *a* of FXIII. The macrophage nature of these cells is clearly demonstrated and by using double- and triple-labelling techniques the FXIII containing subset of TAMs is extensively characterized. Triple labelling systems provide an excellent opportunity to carry out an exact characterization of a cell population, because the direct demonstration of two different antigens in combination with enzyme-cytochemical reactions in the same section yields a precise representation of the coincidence of three different characteristics.

Correspondence: R. Ádány.
Received 21 July 1986; and in revised form, 10 November 1986.

### Materials and methods

Lymph node biopsies were obtained from 12 patients with Hodgkin's disease of nodular sclerosing type. Sections from non-neoplastic, reactive lymph nodes served as controls. All specimens were divided into two parts at the time of surgical biopsy. One part was fixed in 3.5% paraformaldehyde fixative (4 h, room temperature) then vacuum embedded in paraffin and sectioned into 6 μm slides, while the other part was snap-frozen and cut in a cryostat. Six μm frozen sections were air-dried, wrapped in foil and stored at −20°C.

### *Immunoperoxidase staining*

Formaldehyde-fixed paraffin embedded sections were de-waxed and rehydrated. Endogenous peroxidase activity was blocked by 1% $H_2O_2$ in absolute methanol for 30 min at room temperature. Sections were digested with 0.1% trypsin, in TRIS-buffered saline (pH 7.6) containing 0.1% calcium chloride at 37°C for 20 min. Non-specific IgG binding was prevented by preincubation with 20% normal goat serum for 15 min. Sections were covered for 2 h, at room temperature with rabbit antiserum against FXIII subunit *a* (Behringwerke AG, Marburg, West Germany) diluted 1:25 with 20% normal goat serum. The monospecifity of this antiserum was verified by immunoblotting on whole human plasma as well as on human platelet and monocyte homogenate (Muszbek *et al.*, 1985). Antigen-antibody reaction was detected by biotinylated anti-rabbit IgG and avidin-biotinylated peroxidase complex (Vectastain ABC kit) (Vector Laboratories, Burlingame, CA). The specific peroxidase activity was visualized by 0.05% 3,3′-diaminobenzidine tetrahydrochloride (DAB) (Sigma Co., St. Louis, MO), 0.01% $H_2O_2$ in 0.1 mol $l^{-1}$ TRIS HCl buffer, pH 7.2. Counterstaining was with Mayer's haematoxylin before dehydration in graded alcohol and mounting with Canada balsam. On control slides normal rabbit serum at the same dilution was used instead of anti FXIII subunit *a* antiserum. PBS, pH 7.3, was used for antibody dilution and in washing procedures.

*Immunofluorescent and enzyme-histochemical techniques*

Frozen sections were used for the double immunolabelling techniques combined with the cytochemical localization of several enzymes on the same slide (combined triple labelling systems). Immediately before immunoreactions cryostat sections were unwrapped and fixed in acetone at +4°C, for 10 min. After washing in PBS, slides were incubated with 1:200 dilution of antiserum against FXIII subunit *a* for 2 h at room temperature. As the secondary antiserum, a 1:40 dilution of swine anti-rabbit IgG fluoresceinated (Dakopatts a/s, Glostrup, Denmark), was used (30 min incubation). In the next stage this reaction was combined either with the detection of HLA-DR antigen or with the visualization of Leu M3, a monocyte/macrophage surface marker. Sections were incubated with 1:5 dilution of biotinylated mouse anti-human HLA-DR monoclonal antibody (Becton-Dickinson, Sunnyvale, CA) or with the same dilution of mouse anti-human Leu M3 monoclonal antibody conjugated with phycoerythrin (Becton-Dickinson, Sunnyvale, CA) for 30 min, at room temperature. The specific binding of biotinylated anti-HLA-DR antibody was detected by 1:40 dilution of streptavidin-Texas red (Amersham, UK). In the case of negative controls non-immune rabbit serum and control mouse IgG from tumour-bearing BALB/c mice conjugated with phycoerythrin or FITC (Becton Dickinson, Sunnyvale, CA) were substituted for the first antibodies.

Following double immunofluorescent staining, sections were mounted in 50% glycerol in PBS and examined under an Opton ultraviolet microscope equipped with an epifluorescence condensor containing selective filters for FITC and Texas red/phycoerythrin. After photographs had been taken, cover-slips were removed, sections were washed thoroughly in distilled water.

As a third step, one of the following enzymes was detected: α-napthyl acetate esterase (ANAE) (Mueller *et al.*, 1975), acid phosphatase (AcP) (Poulter *et al.*, 1983), adenosine triphosphatase (ATPase) (Poulter *et al.*, 1983) and alkaline phosphatase (ALP) (Mason & Woolston, 1982).

In the case of negative control slides in the enzymecytochemical reactions either the substrate was omitted from the incubation medium, or before development of an enzyme reaction the appropriate enzyme activity was blocked according to the recommendation of Poulter *et al.* (1983). Finally, sections were remounted in 50% glycerol in PBS and the fields of which photographs had been previously taken were identified in normal light microscope and rephotographed. Using the above-mentioned immuno- and enzyme-histochemical reactions all possible combinations of triple labelling were performed on every biopsy specimen.

### Results

Immunoperoxidase staining on paraformaldehyde-fixed, paraffin embedded sections was used to verify the presence and determine the distribution of FXIII subunit *a* containing cells in lymph nodes with Hodgkin's disease of nodular sclerosing type. As demonstrated in Figures 1a, 4b, 5a and 6a practically the whole area of lymph node was infiltrated by cells showing intensive staining for FXIII. These cells were often found in the immediate vicinity of malignant Hodgkin's cells suggesting an intimate relationship between the two cell types (Figure 1b).

FXIII containing-cells in lymph nodes with Hodgkin's disease possess a macrophage-like appearance, they are relatively large, multipolar, mononuclear cells with numerous vacuoles in the cytoplasm. Their detailed characterization was performed by triple labelling (double immunofluorescent + enzyme-cytochemical) techniques. Staining for FXIII and the monocyte/macrophage marker Leu M3 (Dimitriu-Bona *et al.*, 1983) showed an identical distribution pattern in each case (Figure 3a, b). This fact together with their definite ANAE positivity (Figure 4b, c) clearly identifies FXIII containing-cells as members of the macrophage family. These cells were predominantly negative for HLA-DR though occasionally very weak positive staining could also be detected (Figures 2, 4a, b). ALP (Figure 5a, b) and ATPase activities (not shown) could not be detected in FXIII containing cells and surprisingly, they were consistently negative for AcP (Figure 6a, b), as well. The presence of HLA-DR or AcP positive but FXIII negative cells with macrophage-like morphological features clearly indicates that FXIII is expressed only in a certain subset of TAMs.

In contrast to lymph nodes with Hodgkin's disease, in reactive lymph nodes, FXIII containing cells were localized almost exclusively in perivascular connective tissue and in subcapsular or medullary sinuses. Macrophages in follicles of reactive lymph nodes identified by strong ANAE reaction were consistently negative for FXIII subunit *a* and only a few positive cells could be detected in the interfollicular areas (not shown). A detailed characterization of FXIII containing-cells in reactive lymph nodes is published elsewhere (Nemes *et al.*, 1986).

### Discussion

Plasma FXIII, like most other clotting factors circulates as a zymogen. It is a tetrameric protein consisting of two types of subunits ($a_2b_2$). The potential enzymatic site is located on subunit *a* which can assume an active configuration only following proteolytic cleavage by thrombin and $Ca^{2+}$ induced dissociation from the inhibitory *b* subunit (see Muszbek & Laki, 1984 for review). It has been known for a long time that subunit *a* but not *b* also exists as an intracellular protein in platelets (Buluk, 1955), megakaryocytes (Kiesselbach & Wagner, 1972) and placenta (Bohn & Schwick, 1971). As mentioned earlier the existence of subunit *a* in monocytes has also been verified (Muszbek *et al.*, 1985) and these cells retain their FXIII content following differentiation into peritoneal macrophages (Ádány *et al.*, 1985). Furthermore, it was shown that FXIII containing cells in the placenta are also of macrophage origin (Ádány & Muszbek, submitted for publication).

In this study a cell type expressing FXIII subunit *a* was detected in lymph nodes with Hodgkin's disease. The morphological appearance of FXIII containing cells indicated that they belong to TAMs, thus combinations of immuno- and enzyme-histochemical reactions – generally used for identification and/or phenotyping of macrophages – were applied to characterize them. Their macrophage nature was clearly demonstrated by Leu M3 and ANAE positivity. At the same time negative reactions were found for HLA-DR, AcP, ALP and ATPase.

The above results strongly suggest that this FXIII containing-TAM cell type is not identical with any of the macrophage cell types characterized earlier in normal human lymph node. This conclusion is clearly supported by the following data: (i) FXIII containing-macrophages are not identical with interdigitating reticular cells and dentritic cells of lymph node because the latters are strongly positive for HLA-DR and ATPase (Janossy *et al.*, 1980; Stein *et al.*, 1980; Poulter *et al.*, 1983). (ii) They differ from sinus histiocytes of normal lymph node and from inflammatory macrophages, as well as by the absence of AcP activity (Poulter *et al.*, 1983). (iii) Being ALP negative they may not be interpreted as fibroblastic reticulum cells (Chilosi *et al.*, 1981). On the other hand, FXIII containing-cells in lymph nodes with Hodgkin's disease are not even identical with other FXIII containing-cells of normal lymph nodes which occur in the connective tissue of capsule and in the sinus (Nemes *et al.*, 1986). HLA-DR positivity of connective tissue histiocytes as well as the intensive AcP reaction of sinus macrophages clearly distinguish the latter cell types from FXIII containing-TAMs.

**Figure 1** Immunoperoxidase staining for FXIII subunit *a* combined with haematoxylin-chromotrope counterstaining on a section of paraformaldehyde-fixed, paraffin embedded lymph node with Hodgkin's disease, nodular sclerosis type. FXIII subunit *a*-containing cells (in brown colour) invaded practically the whole area of lymph node (a) and were frequently found in the immediate vicinity of malignant Hodgkin's cells (b). Bar = 25 μm (a) and 10 μm (b).

**Figure 2** Double immunofluorescent labelling for FXIII subunit *a* and HLA-DR on a cryostat section of lymph node with Hodgkin's disease. In a double exposure photograph green FXIII containing cells are easily distinguishable from Texas red labelled HLA-DR positive ones. Bar = 10 μm.

**Figure 3** In lymph node with Hodgkin's disease identical cells were labelled with immunofluorescent staining for FXIII subunit *a* (a) and for the monocyte/macrophage surface antigen Leu M3 (b). Bar = 10 μm.

**Figure 4** Triple labelling for HLA-DR (a), FXIII subunit *a* (b) and ANAE (c) on the same area of lymph node with Hodgkin's disease. FXIII containing cells are ANAE positive (see arrows), but HLA-DR negative. Bar = 25 $\mu$m.

Different types of macrophages originate from a common bone marrow precursor (van Furth, 1981) but owing to an intricate differentiation program associated with the expression and disappearance of various gene products (Dimitriu-Bona *et al.*, 1983) they show an extreme phenotypic and functional diversity. In consideration of this fact we believe that FXIII containing-TAMs are a subset of macrophages differentiated from blood monocytes for special function(s) related to malignant cell proliferation. Independently from FXIII containing-macrophages some AcP positive macrophage-like cells were also detected. This finding together with the presence of HLA-DR positive, but FXIII negative cells in lymph nodes with Hodgkin's disease support the possibility that TAMs are to be considered as an inhomogeneous cell population.

Extravascular fibrin deposition occurs in the stroma and at host-tumour interface in most or perhaps all malignant neoplasms (Dvorak *et al.*, 1983). The pathological significance and the origin of fibrin deposits has not been sufficiently explored. Most of the available clinical and experimental data, however, tend to support the view that the activation of clotting system is beneficial for both tumour progression and metastasis formation. Mostly on the basis of theoretical considerations the following hypotheses have been proposed for the pathogenic role of fibrin formed between and around tumour cells: (1) it might have a barrier function and interferes with the host's immune response, (2) it could stimulate tumour angiogenesis, (3) it may have a role in the implantation of circulating tumour cells at metastatic sites (Dvorak *et al.*, 1983). An important role for FXIII – fibrin stabilizing factor – in any of the above mechanisms seems rather obvious. By forming fibrinolysis-resistant fibrin meshwork and crosslinking fibrillar intercellular matrix components, FXIII might support the barrier function or might even be essential to it. The process of angiogenesis and fibroplasia during tumour growth and wound healing has been compared (Dvorak *et al.*, 1983) and it is well known that wound healing is highly impaired in FXIII deficient patients (Duckert, 1972). A possible direct effect of FXIII on cell proliferation, as observed in the case of fibroblasts (Beck *et al.*, 1961), might also have some implications for tumour growth. A further possibility concerns the transglutaminase nature of activated FXIII. As a transglutaminase it can attach host proteins covalently to the membrane of tumour cells and mask their putative 'non-self' character resulting in increased immune resistance. The latter idea is supported by data showing that attachment of fibrinogen to the membrane of YPC-1 plasmocytoma cells by tissue transglutaminase results in an inhibition of cell mediated cytotoxic response (Hunyadi *et al.*, 1981).

In tumours FXIII could get into the interstitial space from two possible sources. It may leak from blood vessels together with other plasma proteins due to enhanced microvascular permeability or it may originate from the TAM subgroup described here by active secretion and/or release following cell destruction. However, this question has not been addressed experimentally. Clearly, further investigations are to be carried out to test the above hypotheses and establish the role of FXIII as well as FXIII containing-TAMs in the progression of malignant tumours.

These studies were conducted in part pursuant to a contract with the National Foundation for Cancer Research, Bethesda, Maryland, USA. We thank Mr Róbert Herendi for the expert technical assistance and Mrs Mária Kozma for the excellent secretarial work.

**Figure 5** Immunofluorescent staining for FXIII subunit *a* (a) combined with the detection of alkaline phosphatase (b) on the same section of lymph node with Hodgkin's disease. As alkaline phosphatase activity was revealed with naphtol AS-MX plus Fast Red TR the end product of the reaction appeared in red colour not only in normal light but on the FITC channel of the fluorescence microscope, as well. On the photograph it is superimposed on FITC labelled immunreaction for FXIII subunit *a*. FXIII containing macrophages are negative for alkaline phosphatase. Bar = 25 $\mu$m.

**Figure 6** In lymph node with Hodgkin's disease immunofluorescent staining for FXIII subunit *a* (a) and acid phosphatase activity detection (b) visualize two distinct cell populations. Bar = 25 $\mu$m.

### References

ÁDÁNY, R., BELKIN, A., VASILEVSKAYA, T. & MUSZBEK, L. (1985). Identification of blood coagulation factor XIII in human peritoneal macrophages. *Eur. J. Cell Biol.*, **38**, 171.

BECK, E., DUCKERT, F. & ERNST, M. (1961). The influence of fibrin stabilizing factor on the growth of fibroblasts *in vitro* and wound healing. *Thromb. Diath. Haemorrhag.*, **6**, 485.

BOHN, H. & SCHWICK, H.G. (1971). Isolierung und Charakterisierung eines fibrinstabilisierenden Faktors aus menschlichen. *Plazenten. Arzneim. Forsch.*, **21**, 1432.

BULUK, K. (1955). An unknown function of blood platelets. *Polski Tygod. Lekar.*, **10**, 191.

CHAPMAN, H.A., ALLEN, L.C. & STONE, O.L. (1985). Human alveolar macrophages synthesize factor VII *in vitro*. *J. Clin. Invest.*, **75**, 2030.

CHILOSI, M., PIZZOLO, G., MENESTRINA, F., IANNUCCI, A.M., BONETTI, F. & FIORE-DONATI, L. (1981). Enzyme histochemistry on normal and pathologic paraffin-embedded lymphoid tissues. *Am. J. Clin. Pathol.*, **76**, 729.

DIMITRIU-BONA, A., BURMESTER, G.R., WATERS, S.J. & WINCHESTER, R.J. (1983). Human mononuclear phagocyte differentiation antigens. I. Patterns of antigenic expression on the surface of human monocytes and macrophages defined by monoclonal antibodies. *J. Immunol.*, **130**, 145.

DUCKERT, F. (1972). Documentation of the plasma factor XIII deficiency in man. *Ann. N.Y. Acad. Sci.*, **202**, 190.

DVORAK, H.F., SENGER, R.D. & DVORAK, A.M. (1983). Fibrin as a component of the tumor stroma: origins and biological significance. *Cancer Metast. Rev.*, **2**, 41.

EDWARDS, R.L., RICKLES, E.R. & CRONLUND, M. (1981). Abnormalities of blood coagulation in patients with cancer: mononuclear cell tissue factor generation. *J. Lab. Clin. Med.*, **98**, 917.

EVANS, R. (1982). Macrophages and neoplasms: new insights and their implication in tumor immunobiology. *Cancer Metast. Rev.*, **1**, 227.

GORDON, S.G. & CROSS, B.A. (1981). A factor X-activating cystein protease from malignant tissue. *J. Clin. Invest.*, **67**, 1665.

GORDON, S.G., FRANKS, J.J. & LEWIS, B.J. (1975). Cancer procoagulant A: a factor X activating procoagulant from malignant tissue. *Thrombos. Res.*, **61**, 127.

GORDON, S.G., FRANKS, J.J. & LEWIS, B.J. (1979). Comparison of procoagulant activities in extracts of normal and malignant human tissue. *J. Natl. Cancer Inst.*, **62**, 773.

HARRIS, N.L., DVORAK, A.M., SMITH, H. & DVORAK, H.F. (1982). Fibrin deposits in Hodgkin's disease. *Am. J. Pathol.*, **108**, 119.

HANSMANN, M.L. & KAISERLING, E. (1981). Electron-microscopic aspects of Hodgkin's disease. *J. Cancer Res. Clin. Oncol.*, **101**, 135.

HENRIKSSON, P., BECKER, S., LYNCH, G. & McDONAGH, J. (1985). Identification of intracellular factor XIII in human monocytes and macrophages. *J. Clin. Invest.*, **76**, 528.

HOPPER, K.E., WOOD, P.R. & NELSON, D.S. (1979). Macrophage heterogeneity. *Vox. Sang.*, **36**, 257.

HUNYADI, J., SZEGEDI, G., SZABÓ, T., AHMED, A. & LAKI, K. (1981). Increased cytotoxic sensitivity of YPC-1 tumor cells from mice treated with nitrosoureas. *Cancer Res.*, **41**, 1677.

JANOSSY, G., THOMAS, J.A., PIZZOLO, G. & 5 others. (1980). Immunohistological diagnosis of lymphoproliferative diseases by selected combinations of antisera and monoclonal antibodies. *Br. J. Cancer*, **42**, 224.

KEY, M. (1983). Macrophages in cancer metastases and their relevance to metastatic growth. *Cancer Metast. Rev.*, **2**, 75.

KIESSELBACH, T.H. & WAGNER, R.H. (1972). Demonstration of factor XIII in human megakariocytes by a fluorescent antibody technique. *Ann. N.Y. Acad. Sci.*, **202**, 318.

LINDAHL, U., KOLSET, S.O., BØGWALD, J., ØSTERUD, B. & SELJELID, R. (1982). Studies with a luminogenic substrate on blood coagulation factor X/Xa produced by mouse peritoneal macrophages. *Biochem. J.*, **206**, 231.

LORENZET, R., PERI, G., LOCATI, D. & 5 others. (1981). Generation of procoagulant activity by mononuclear phagocytes: a possible mechanism contributing to blood clotting activation within malignant tissues. *Blood*, **62**, 271.

MASON, D.Y. & WOOLSTON, R.E. (1982). Double immunoenzymatic labelling. In *Techniques in Immunocytochemistry*. Bullock, G.R. & Petrusz, P. (eds) **Vol. 1**, p. 135. Academic Press: New York.

MUSZBEK, L., ÁDÁNY, R., SZEGEDI, G., POLGÁR, J. & KÁVAI, M. (1985). Factor XIII of blood coagulation in human monocytes. *Thrombos. Res.*, **37**, 401.

MUSZBEK, L. & LAKI, K. (1984). Interaction of thrombin with proteins other than fibrinogen (thrombin susceptible bonds). Activation of factor XIII. In *The Thrombin*, Machovich, R. (ed) p. 83. CRC Press: Boca Raton.

MUELLER, J., BRUN DEL RE, G., BUERKI, H., KELLER, H.K., HESS, M.W. & COTTIER, H. (1975). Nonspecific acid esterase activity a criterion for differentiation of T and B lymphocytes in mouse lymph nodes. *Eur. J. Immunol.*, **5**, 270.

NEMES, Z., THOMÁZY, V., ÁDÁNY, R. & MUSZBEK, L. (1986). Identification of histiocytic reticulum cells by the immunohistochemical demonstration of factor XIII (F-XIIIa) in human lymph nodes. *J. Pathol.*, **149**, 121.

O'MEARA, R.A.Q. (1958). Coagulative properties of cancers. *Irish J. Med. Sci.*, **6**, 474.

ØSTERUD, B., LINDAHL, U. & SELJELID, R. (1980). Macrophages produce blood coagulation factors. *FEBS Letters*, **120**, 41.

POULTER, L.W., CHILOSI, M., SEYMOUR, G.J., HOBBS, S. & JANOSSY, G. (1983). Immunofluorescence membrane staining and cytochemistry, applied in combination for analysing cell interactions *in situ*. In *Immunocytochemistry. Practical applications in pathology and biology*, Polak, J.M. & Van Noorden, S. (eds) p. 233. Wright PSG: Bristol.

RICKLES, E.R. & EDWARDS, R.L. (1983). Activation of blood coagulation in cancer: Trousseau's syndrome revisited. *Blood*, **62**, 14.

SCHLAGER, S.I. & DRAY, S. (1975). Complete tumor regression with antibody to fibrin fragment E. *J. Immunol.*, **115**, 976.

SEMERARO, N. & DONATI, M.B. (1981). Pathways of blood clotting initiation by cancer cells. In *Malignancy and the hemostatic system*, Donati, *et al.*, (eds) p. 65. Raven Press: New York.

STEIN, H., BONK, A., TOLKSDORF, G., LENNERT, K., RODT, H. & GERDES, J. (1980). Immunohistologic analysis of the organization of normal lymphoid tissue and non-Hodgkin's lymphomas. *J. Histochem. Cytochem.*, **28**, 746.

STILLER, D. & KATENKAMP, D. (1978). Intracellular substances in Hodgkin's lymphomas. Ultrastructural investigations. *Virchows Arch. A. Path. Anat. Histol.*, **380**, 81.

VAN DAM-MIERAS, M.C.E., MULLER, D.A., VAN DEIJK, W.A. & HEMKER, H.C. (1985). Clotting factors secreted by monocytes and macrophages: analytical considerations. *Thrombos. Res.*, **37**, 9.

VAN FURTH, R. (1981). Identification of mononuclear phagocytes: overview and definitions. In *Methods for studying mononuclear phagocytes*, Adams, *et al.* (eds) p. 243. Academic Press: New York.